# Editorial: The application of OMICS technologies to interrogate host-virus interactions

**DOI:** 10.3389/fcimb.2022.1050012

**Published:** 2022-11-14

**Authors:** Fabio Gomes, Kendra Alfson, Magno Junqueira

**Affiliations:** ^1^ Instituto de Biofísca, Universidade Federal do Rio de Janeiro, Rio de Janeiro, Brazil; ^2^ Department of Disease Intervention and Prevention, Texas Biomedical Research Institute, San Antonio, TX, United States; ^3^ Instituto de Química, Universidade Federal do Rio de Janeiro, Rio de Janeiro, Brazil

**Keywords:** host-virus interaction, mass spectrometry, proteomics, transcriptomics, metabolomics, genomics, biomolecules quantification

Viruses can infect all types of life forms, ranging from humans to bacteria. High-content data generated with omics technologies can be used to identify emergent properties of these systems, providing targets for further mechanistic investigations. Since the completion of the first genome sequencing projects, omics approaches have been used to study the dynamics and complexity of host-virus interactions. Starting with early microarray studies aiming to cover a fraction of the host genome and progressively moving into deep-sequencing projects, these studies shed light on the modulation of host gene expression profiles upon infection. In addition, changes in the protein level could be accessed *via* mass spectrometry-based proteomic methods (Luo and Muesing 2014), applied on a genome scale [reviewed in (Lum and Cristea, 2016)]. These investigations allowed direct protein quantification that partially validated previous transcriptomic findings and highlighted the complexity of the regulation of protein translation and post-translation modifications during virus infection (Hoogendijk et al., 2019; Kumar et al., 2020). More recently, network analysis has been progressively used to identify promising targets for therapeutic interventions and drug repurposing, and is now playing a role in vaccine development studies (Hagan et al., 2015; Pulendran et al., 2021). At the same time, reduced sequencing costs and evolving hardware capabilities now allow for massive projects involving multi-omics data integration (Sammut et al., 2022) and reviewed in (Wang et al. 2019; Appiasie et al., 2021). Therefore, this Research Topic discloses the state-of-the-art omics technology applied to virus-host interactions. It introduces five selected articles from leading groups, covering a selection of up-to-date subjects revealing the complexity and diversity of omics technology.

## Models and markers of disease progression

While *in vitro* models are invaluable tools for the research of cell-virus interactions, including the development of high-throughput screenings and live cell imaging assays, robust animal models are imperative for the understanding of immunological, physiological and metabolic impacts of virus infections. Nonhuman primates are an important alternative to murine models of arboviral diseases, as several viruses, including dengue virus (DENV) and Zika virus (ZIKV) usually fail to replicate in mice. Mask et al. investigated the molecular signature of ZIKV in baboons. They showed that, like infection in humans, ZIKV infection in baboons usually results rapidly in subclinical cases, with a transient antiviral interferon-based response signature. The similarity between human and baboon infection suggests that the latter could be used as a model for the investigation of the molecular basis of ZIKV infections, including predicting the early molecular markers of case aggravation. Among others, molecular markers are paramount for predicting disease progression and aggravation. Moraes et al. used label-free shotgun proteomics to investigate the role of extracellular vesicles (EV) in the pathology of coronavirus disease of 2019 (COVID-19). They observed an increase in the abundance of EVs in patients with severe COVID-19 and identified proteins involved with complement and coagulation pathways, platelet degranulation, and acute inflammatory response that might serve as markers of severe COVID-19, as well as help explain the pathological pathways involved with disease aggravation.

## Disease transmission by insect vectors

Arboviral diseases use insect vectors as a vehicle for virus dissemination. In that sense, vectors must be able to allow the virus to infect, replicate and colonize target tissues before being transmitted to the host following a blood meal – a potential that is influenced by several factors, collectively known as vector capacity. The interaction of viruses with their cellular host triggers an immunometabolic response that must be circumvented by the virus during its replication cycle. However, knowledge concerning how mosquito cells respond to arboviral infections and how viruses evade this immune response remains scant. Vasconcellos et al. studied the proteomic profile of *A. aegypti*-derived Aag2 cells infected with chikungunya virus (CHKV). They identified 196 regulated protein groups upon infection, which are related to protein synthesis, energy metabolism, signaling pathways, and apoptosis, narrowing a list of proteins that could be associated with antiviral and/or proviral mechanisms and the balance between viral propagation and the survival of host cells. Zika virus is an arbovirus that can cause disease associated with negative fetal outcomes such as microcephaly (reviewed in (Masmejan et al., 2020). A control strategy relies on introducing *Ae. aegypti* mosquitoes carrying *Wolbachia pipientis* to the environment, as these mosquitoes are less susceptible to arbovirus infection and have reduced fertility. Ramos et al. describes effects of ZIKV and *Wolbachia* on reproduction and the immune system in the *Ae. aegypti*, using isobaric-labeled quantitative proteomics. Mass spectrometry-based proteomics are highly effective for assessing host-pathogen interactions (also reviewed in (Sivanich et al., 2022). Isobaric labeling quantitative methods allow for multiplexing, which allows for: higher throughput, increased statistical robustness, sample conservation, high coverage depth, and increased efficiency. This research resulted in several crucial findings, about the impact these microorganisms have on the vector reproduction and immune system. The authors provide a thorough discussion of the *Ae. aegypti* ovary proteome; many *Wolbachia* proteins; and numerous proteins that were modulated during infection (both mono and coinfections), in order to investigate which pathways were altered. Importantly, they identified that Juvenile Hormone pathway is modulated by both ZIKV and *Wolbachia*; ZIKV may enhance vector reproduction while *Wolbachia* seems to be harmful. Further, ZIKV seems to facilitate infection, while *Wolbachia* blocks infection and enhances immune priming. This work can be an important resource for understanding how microorganism infection can influence *Ae. aegypti* immune response and reproducibility. These fascinating results show the utility of isobaric labeling-based quantitative proteomics in investigating host-pathogen interactions and can be used to better design control strategies.

## The epitranscriptomics of infected cells

During viral infection, not only do the RNA levels of the host cell change, but several RNA nucleotide modifications also occur. Campos et al. tested the hypothesis that infection of Vero cells by Severe acute respiratory syndrome coronavirus 2 (SARS-CoV-2) affects the m6A methylation patterns of cellular transcripts that play important roles in regulating gene expression. For this, the epitranscriptome of the infected cell was sequenced using the Nanopore direct RNA sequencing method. Datasets from four studies were compared and revealed that the m6A methylation of cellular RNAs is higher in infected cells. This paper represents a valuable data resource for epitranscriptome changes regulating SARS-CoV-2 infection.

## Author contributions

FG, KA, and MJ contributed to development of the Research Topic, editorial and reviewed this manuscript. MJ contributed to the idealization, development of the Research Topics and editorial, and prepared the draft and final version of this manuscript. All authors contributed to the article and approved the submitted version.

## Funding

This work was supported by Fundação de Amparo à Pesquisa do Estado do Rio de Janeiro - FAPERJ - Grant: Temáticos FAPERJ E-26/211.348/20 and by Conselho Nacional de Desenvolvimento Científico e Tecnológico- CNPq.

## Conflict of interest

The authors declare that the research was conducted in the absence of any commercial or financial relationships that could be construed as a potential conflict of interest.

## Publisher’s note

All claims expressed in this article are solely those of the authors and do not necessarily represent those of their affiliated organizations, or those of the publisher, the editors and the reviewers. Any product that may be evaluated in this article, or claim that may be made by its manufacturer, is not guaranteed or endorsed by the publisher.
